# Twinning and homo-epitaxy cooperation in the already rich growth morphology of CaCO_3_ polymorphs. II. Calcite

**DOI:** 10.1107/S1600576724008057

**Published:** 2024-09-20

**Authors:** Dino Aquilano, Stefano Ghignone, Marco Bruno

**Affiliations:** a Dipartimento di Scienze della Terra Università degli Studi di Torino Via Valperga Caluso 35 10125Torino Italy; b NIS, Centre for Nanostructured Interfaces and Surfaces Università degli Studi di Torino Via G. Quarello 15/a 10135Torino Italy; Tohoku University, Japan

**Keywords:** calcite, twinning, homo-epitaxy, growth morphology

## Abstract

The two most abundant CaCO_3_ polymorphs, calcite and aragonite, are universally recognized for the richness of their morphology to which different twins make relevant contributions. The epitaxial transformation calcite ↔ aragonite has long been debated. While the twinning has been thoroughly treated, the homo-epitaxy occurring within each of these minerals has, inexplicably, been overlooked to date, both experimentally and theoretically. Twinning can be deceptive to the point where it can be mistaken for homo-epitaxy, thus making the proposed growth mechanism in the crystal aggregate wrong. Within the present work, the first aim is a theoretical investigation of the homo-epitaxies among the three {10.4}-cleavage, {01.2}-steep and {01.8}-flat rhombohedra of calcite. Accordingly, the specific adhesion energies were calculated between facing crystal forms, unequivocally showing that the {01.2}/{01.8} homo-epitaxy competes with the generation of both {01.2} and {01.8} contact twins. Secondly, the calculation of the specific adhesion energy was extended to consider homo-epitaxy for the {10.4} rhombohedron. The two-dimensional geometric lattice coincidence has been tried for the {00.1} pinacoidal form as well.

## Abstract 2

A theoretical investigation is presented of the homo-epitaxies among the three {10.4}-cleavage, {01.2}-steep and {01.8}-flat rhombohedra of calcite.

## 1. Introduction

Extended and operative definitions of twinning, epitaxy, endotaxy and topotaxy were given many years ago in the careful lectures by Kern (1989 ▸, 1996 ▸), who recollected the historical and fundamental papers by Friedel (1926 ▸) and Royer (1928 ▸). These definitions have been refined very recently in Part I of this series (Aquilano *et al.*, 2023 ▸).

As extensively demonstrated on aragonite (Aquilano *et al.*, 2023 ▸), the novelty that we introduced in Part I was the clarification of the term ‘homo-epitaxy’. This term will also be used in the present paper for calcite, the other most commonly occurring CaCO_3_ polymorph (space group 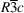, *a*_0_ = *b*_0_ = 4.9896 Å, *c*_0_ = 17.061 Å) (Bruno *et al.*, 2010 ▸). We will explain that, in the same crystal species (*A*), two or more different forms {*hkl*} and {*h*′*k*′*l*′} can associate through an epi-relation without producing a new twin law. This suggests a requirement to be very careful in researching the relationship between two different {*hkl*} forms of the same substance *A* and to always check whether there is an original composition plane or symmetry axis (which does not belong to the *A* symmetry) intervening between the aforementioned forms. Following such a definition, we will first investigate calcite (hereinafter abbreviated to Cal) at an empirical level and demonstrate that both steep {01.2} and flat {01.8} rhombohedra can be homo-epitaxially related. Secondly, we will show that the {10.4} rhombohedron and the basal {00.1} pinacoid also enter homo-epitaxy with these {01.2} and {01.8} forms. Very recently Németh *et al.* (2018 ▸) observed {01.2}/{01.8} homo-epitaxy but without interpreting it. On the other hand, it has been shown that the {01.2} and {01.8} forms give rise to well known twin laws, although there cannot be any twin correlation between them (Bruno *et al.*, 2010 ▸).

We recently reported that the hexaragonite phase originates, at room temperature and pressure, from aragonite through a homo-epitaxial mechanism (Bruno *et al.*, 2022 ▸), making this is a timely occasion to enter the long-standing debate about the calcite ↔ aragonite transformation (Brar & Schloessin, 1979 ▸, 1980 ▸; de Leeuw & Parker, 1998 ▸; Sekkal & Zaoui, 2013 ▸; Bruno *et al.*, 2022 ▸).

For two distinct crystal forms *A* and *B*, one has to distinguish homo-epitaxy (*A*/*A* → homo-epi) from both twinning (*A*/*A* → twins) and hetero-epitaxy (*A*/*B*). Homo-epitaxy differs from the other two, obviously, in terms of geometry (lattices) and also for the prominent role exerted by the physical chemistry; hence, the homo-epitaxial relationships are not entirely governed by the geometry but will be affected, as for aragonite, by the specific adhesion energy of the facing crystal forms.

## 2. Computational method

To study the homo-epitaxial relationships in calcite, here we investigated the epi-interfaces at an empirical level, determining both their structures and their thermodynamic properties at 0 K. A composed calcite slab, (*hkl*)/(*h*′*k*′*l*′), was generated (Bruno *et al.*, 2015 ▸, 2017 ▸) in the following way:

(i) We searched for the two-dimensional coincidence lattices (2D-LCs hereinafter) between the (*hkl*) and (*h*′*k*′*l*′) faces of calcite, in epitaxial relationship at reticular level, and subsequently considered only those fulfilling rigorous epitaxy constraints.

(ii) (*hkl*) and (*h*′*k*′*l*′) slabs of a selected thickness were made by cutting the bulk structure of calcite parallel to the lattice planes of interest and using the 2D-LC parameters describing the found epitaxy.

(iii) The (*hkl*) slab was placed above the (*h*′*k*′*l*′) slab.

(iv) Finally, the composed slab structures (atomic coordinates and 2D-LC parameters) were optimized by considering all of the atoms free to move.

The epitaxy constraints applied here follow the rigorous set of limitations that we imposed in our preceding work, in order to prevent erroneous identification of 2D-LCs, *i.e.* linear and area misfits <10%, along with an angular misfit <5°. A recent example can be found in the report by Pastero & Aquilano (2018 ▸).

Structure optimization of the (01.8)/(01.2), (10.4)/(01.2) and (10.4)/(01.8) composed calcite slabs has been performed at empirical level using the calcium carbonate force field (Rohl *et al.*, 2003 ▸) and version 4.0 of the *GULP* simulation code (Gale, 1997 ▸). The computational parameters we adopted are suitable to guarantee convergence on the energy values discussed below, as well as the thickness of the composed slab. The *GULP* output files, listing the optimized fractional coordinates along with the optimized 2D-LC parameters, are freely available at https://marco-bruno.weebly.com/download.html. We only performed static calculations at 0 K, the vibrational entropy and energy not being calculated. However, as previously discussed (Bruno *et al.*, 2013 ▸; Bruno, 2015 ▸), neglecting the vibrational contribution should not lead to a significant error in the estimate of the adhesion and interfacial energies. A detailed description of the computational method­ology used for the interfaces has already been reported (Bruno *et al.*, 2015 ▸, 2017 ▸).

The adhesion energy 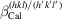 (in units of erg cm^−2^) reads
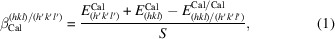
where 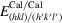, 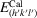 and 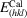 represent the energies of the composed (*hkl*)/(*h*′*k*′*l*′) and isolated (*h*′*k*′*l*′), (*hkl*) slabs, respectively, while *S* is the area of the 2D-LC. 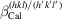 is related to the specific interface energy 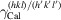 (erg cm^−2^) by the Dupré relation (Kern, 1978 ▸),
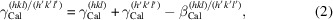
where 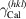 and 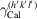 are the specific surface energies in a vacuum of the (*hkl*) and (*h*′*k*′*l*′) faces, respectively.

## 3. Results and discussion

### 3.1. Selected twinning in calcite

Within this section, we recollect the relationships between a given interfacial twin energy 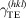 and the corresponding specific adhesion energy β^(*hkl*)/(*hkl*)^ recovered, once the twin has been formed. The specific 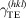 value is the energy to be spent per unit area of a given (*hkl*) face in order to form a twin on it. Looking at the four usually accepted twin laws of calcite, indicated in order of decreasing energy, the following were found (Bruno *et al.*, 2010 ▸): 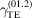 = 259, 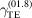 = 183, 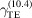 = 162 and 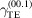 = 1 erg cm^−2^, respectively. To determine the β^(*hkl*)/(*hkl*)^ values for a given *hkl* calcite twin law, built by parent (P) and twinned (T) individuals, the Dupré formula (Friedel, 1926 ▸) reads
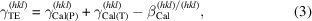
where 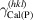 = 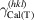 = 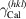 are the specific surface energy of the (*hkl*) face (before twinning). Consequently, equation (3) reduces to
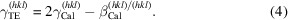


All energies being expressed in erg cm^−2^, and inserting in equation (4) the surface energies 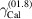 = 702, 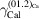 = 1040 and 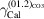 = 750 (Bruno *et al.*, 2008 ▸, 2010 ▸), the adhesion energies for the (01.8) and (01.2) twin laws are 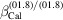 = 1221, 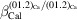 = 1821 and 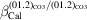 = 1241. The form {01.8} has only one termination, so only one surface energy value is obtained. In contrast, the {01.2} form can be either Ca- or CO_3_-terminated and thus two surface energies are allowed (Bruno *et al.*, 2008 ▸, 2010 ▸). A new epitaxy concerning the {01.2}_Cal_ form, which must be mentioned here, has very recently been found by Aquilano *et al.* (2024 ▸).

### 3.2. The calcite {01.8}/{01.2} homo-epitaxy

The maximum value of the percent misfit (Δ*d*_max_%) between the thickness of the elementary layer *d*_01.2_ (3.8548 Å) and the double layer 2*d*_01.8_ (3.8468 Å) is very low and reaches Δ*d*_max_% = 0.21.

The steep {01.2} rhombohedron has flat (F) character and the steps *d*_01.2_ are self-consistent. However, the flat {01.8} rhombohedron is stepped (S) and then the simple step (*d*_01.8_) is not self-consistent. Thus, double steps (2*d*_01.8_) are needed, generated either through 2D nuclei or by screw dislocations outcropping on the crystal substrate (Hartman, 1987 ▸).

**Figure 1:**
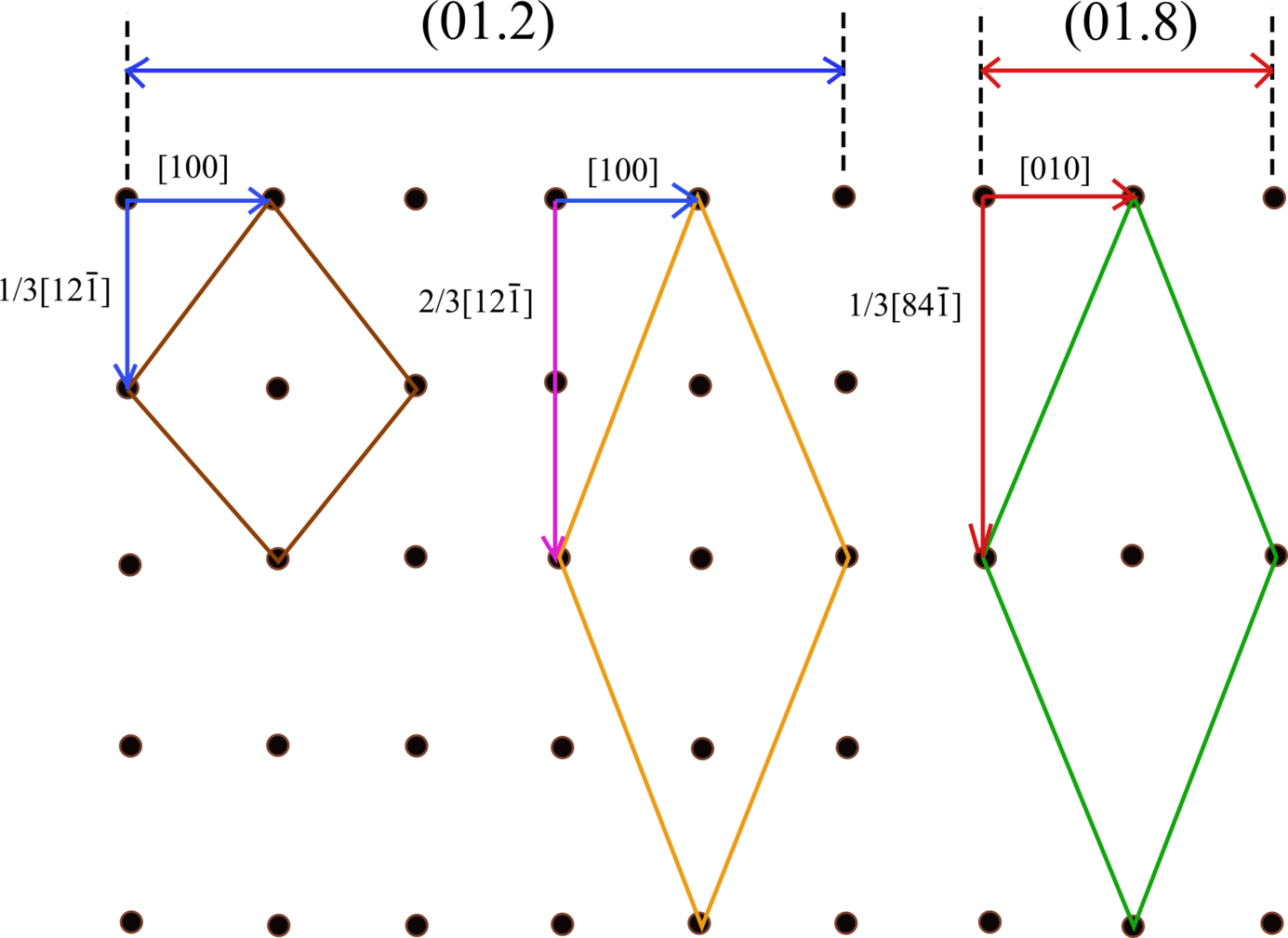
The 2D-LC meshes on the {01.2} and {01.8} forms of calcite, taken from the homo-epitaxy of Table 1. One can see that the vector ⅔ × [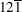] of the {01.2} form is twice the basic ⅓ × [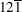]. Moreover, it is obvious that the vectors ⅔ × [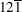] and [100] of the {01.2} macro-cell (orange) nearly equal the vectors ⅓ × [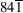] and [010] of the {01.8} macro-cell (red). In such a way, we show that the rectangular cells indicated in Rank 1*a* of Table 1 ▸ are practically identical.

For the sake of simplicity, Table 1 ▸ and Fig. 1 ▸ report only the 2D-LCs used to calculate the 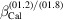 value.

With regard to 2D-LCs, the linear and area misfits concerning the lattices considered here are defined as the maxima (per cent) of the differences between the two lattices. As trivial examples, from Table 1 ▸ one finds % linear misfit = (8.6422 − 8.1030)/8.1030 = 6.65 and % area misfit = (63.61 − 64.12)/64.12 = −0.79. The angular misfit (°) vanishes for rectangular-shaped 2D-LCs, while it obviously differs from zero for lozenge-shaped 2D-LCs.

Let us now consider the peculiar case of the {01.8}/{01.2} calcite homo-epitaxy shown in Fig. 1 ▸. The cost of the best homo-epi interfacial energy 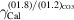 reaches 526 erg cm^−2^ (with 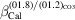 = 926 erg cm^−2^) for the CO_3_-terminated (01.2) surface. This value shows that all of the known calcite twins are always cheaper than the calcite homo-epitaxy we just proposed, since all twin energies of calcite (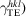 are ≤259 erg cm^−2^. Instead, the homo-epi interfacial energy when considering the Ca-terminated (01.2) surface is higher, being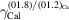 = 1019 erg cm^−2^ and 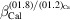 = 723 erg cm^−2^.

Concerning the calcite (01.8)/(01.2) homo-epitaxy, owing to the splitting of the (01.2) surface in two ways, two different adhesion and interface energies were calculated, having adopted the smallest 2D-LC (Table 1 ▸). The minimum value of the homo-epitaxy interface energy is then 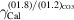 = 526 erg cm^−2^. This is lower than the minima of the surface energies for the isolated {01.2} and {01.8} forms, which are 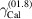 = 702 erg cm^−2^ and 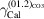 = 750 erg cm^−2^.

Summing up:

(i) The {01.2} and {01.8} twin laws are the most commonly occurring ways of finding these two rhombohedra, with 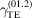 = 259 erg cm^−2^ and 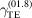 = 183 erg cm^−2^ for calcite, respectively (Aquilano *et al.*, 2023 ▸).

(ii) The specific interfacial energy 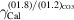
 = 526 erg cm^−2^, surprisingly, competes with the appearance of the isolated and hence not twinned {01.2} and {01.8} rhombohedra.

With regard to the morphological importance (MI), one can state the true novelty of the first part of the present work: single [{01.2}, {01.8}] forms << {01.2}/{01.8} homo-epitaxy ≤ [{01.2}, {01.8}] twins. In other words, from the MI it turns out that {01.2}/{01.8} homo-epitaxy is more common than the existence of {01.2}, {01.8} single forms.

### 3.3. The calcite {01.2}/{10.4} and {01.8}/{10.4} homo-epitaxies

We now introduce the second novelty of this work: not only do the {01.2} and {01.8} rhombohedra participate in calcite homo-epitaxy, but so does the well known {10.4} cleavage rhombohedron.

In the present paper, we considered as useful for the homo-epitaxies only those 2D-LCs giving rise to rectangular 2D supercells. This practice has already been established with aragonite (Aquilano *et al.*, 2023 ▸) where homo-epitaxy was ascertained to exist between its {010} and {110} forms belonging to the [001] zone development.

However, where calcite is concerned, one has to recollect that all its faces (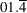), (01.2), (01.8), (010) and (001) have the direction [100] as a common zone axis. Moreover, the calcite face (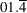) is symmetry equivalent to (10.4), because both of them belong to the same {10.4} crystal form. Nevertheless, the {010} prism and {001} pinacoid cannot participate in this last homo-epitaxy.

In fact, apart from the common vector [100] (or its equivalent [010]), the other vectors defining the respective rectangular 2D cells do not give any 2D-LCs with the (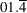), (01.2) and (01.8) faces, since neither their length nor their obliquity satisfies the already established practice valid for aragonite.

Instead, it was a pleasant surprise to note that, introducing the form {10.4} in this homo-epitaxy, the area multiplicity of {01.8} is (2×) and that of {01.2} reaches only (4×) with respect to those we observed. The long-sized moduli of the rectangular 2D-LCs are (Table 1 ▸) 4/3 × [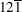] = 25.4988 Å for {01.2}, 2/3 × [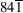] = 25.6996 Å for {01.8} and [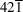] = 24.309 Å for the {10.4} form. Interestingly, the corresponding mean value of these long-sized moduli is 25.169 ± 0.613 Å, where the standard deviation (σ = 0.613) represents only 2.44% of the mean value.

We believe these values are much more sensible than the quoted ones associated with the area misfits: in the area misfits the data are distorted since the short-sized moduli of the 2D-LCs (Table 1 ▸) are always the same, *i.e.* 4.9896 Å, for all the rhombohedral {01.2}, {01.8} and {10.4} forms. For this reason, we considered the calculation of the specific interface energy [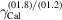] and specific adhesion energy [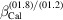] to be broadly sufficient to prove the (01.8)/(01.2) homo-epitaxy. In close analogy, the extension of the homo-epitaxy to the {10.4} form seems reasonable, and hence we calculated 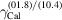
 = 1047 [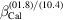
 = 189], 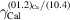
 = 1182 [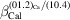
 = 392] and 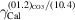
 = 1649 [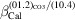 = −365] (in erg cm^−2^). All of the γ_Cal_ values are calculated by considering 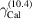 = 534 erg cm^−2^ (Bruno *et al.*, 2013 ▸).

### 3.4. Why homo-epitaxies can involve the three {10.4}, {01.2} and {01.8} rhombohedral and {00.1} pinacoidal forms of calcite, while the {01.0} prism should be excluded

In order to discuss the possible homo-epitaxies involving the {00.1} and {01.0} forms with the three rhombohedra previously discussed, one has to consider two new long-sized moduli: the length (or multiple) of the vector [210] = 8.6422 Å and the length of the vector [001] = 17.0610 Å. These lengths have to be compared with the mean value (25.169 ± 0.613 Å) of the long-sized moduli 4/3 × [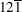], 2/3 × [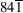] and [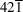], as we reported in Table 1 ▸ for the {01.2}, {01.8} and {10.4} rhombohedra.

As concerns the pinacoid {00.1}, one has to compare the mean value of the long-sized moduli (25.169 ± 0.613 Å) with the vector 3 × [210] = 25.9267 Å; the corresponding percent difference is very low (Δ% = 3.01). For the 2D-LC areas, one needs 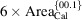 = 129.36 Å^2^ in order to compensate: 2 × 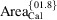
 = 128.23 Å^2^, 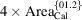
 = 127.23 Å^2^ and 3 × 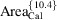
 = 121.29 Å^2^. The percent area misfits become Δ% = 0.88, 1.67 and 6.65, respectively.

A simpler way of approaching the homo-epitaxy between the forms {10.4} and {00.1} of calcite is to avoid the vector of the long-sized modulus, as reported in Table 1 ▸. Accordingly, the difference for both cell parameters and 2D-LC areas increases to Δ% = 6.65, even though the absolute mean area only reaches a value of 41.78 Å^2^.

For the prismatic calcite form {01.0}, we compare its long-sized modulus *n* × [001] = *n* × 17.061 Å with an integer multiple of the mean value of the long-sized moduli we have just calculated, *m* × 25.169 ± 0.613 Å. It is easily shown that 3 × 17.061 Å = 51.183 Å ≃ 2 × 25.169 Å = 50.338 Å. The cor­responding Δ% = 1.68 also reaches a very low misfit value. Nevertheless, the minimum area for a perfect rectangular 2D-LC is equivalent to 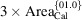 = [010] × 3[001]_Cal_ = 255.38 Å^2^, which is practically twice the values of the best areas we indicated above.

Summing up, and specifically referring to the areas of the 2D-LCs, it is plain that:

(i) The {01.8}/{01.2} homo-epitaxy is widely assured by the very low 2D-LC = 63.86 Å^2^. The corresponding adhesion energy has a high value of 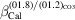 = 926 erg cm^−2^.

(ii) Introducing the cleavage {10.4} form of calcite plus its {00.1} pinacoid would increase the area of the 2D-LC from very low to low values, resulting in a new mean value of 2D-LC = 127.73 Å^2^. Doubling the 2D-LC area means that the 2D-LCs are perfect and moderate, but also that the adhesion energy calculation becomes more complicated and hence less easy to do.

(iii) When going to the prismatic {01.0} form, the minimum area for a perfect rectangular coincidence reaches the above value of 255.38 Å^2^, thus doubling once again the 2D-LC area. At this point, the arguments on the adhesion energy calculation are even more valid.

## 4. Conclusions

The two most common calcium carbonates, calcite and aragon­ite, have long been known to show a very rich production of twins. As recently done for aragonite (Aquilano *et al.*, 2023 ▸), here we have certified that not only does twinning characterize calcite but also there exists a growth mechanism capable of generating a special kind of epitaxy within the same crystalline phase, which we call homo-epitaxy. Twinning and homo-epitaxy resemble each other, even though they are not symmetry-equivalent operations. Moreover, the thermodynamic quantity that unites and distinguishes them is the specific adhesion energy between the facing crystal phases.

Thanks to the homo-epitaxy energy, we were able to use the morphological importance to say that the calcite {01.2}/{01.8} homo-epitaxy (i) can compete with both {01.2} and {01.8} twins and (ii) is more probable than the occurrence of single {01.2} and {01.8} rhombohedra. Hence, and in analogy with our recent paper on aragonite, in calcite the single [{01.2}, {01.8}] forms << {01.2}/{01.8} homo-epitaxy ≤ [{01.2}, {01.8}] twins.

According to our observations, the form {10.4} should participate in the calcite homo-epitaxy, since the interfacial energies 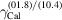
 = 1047 erg cm^−2^ and 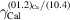
 = 1182 erg cm^−2^ represent practically twice the value of 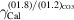
 = 523 erg cm^−2^. Obviously, these calculated values of the interface energy do not allow the cleavage {10.4} rhombohedron to share the just-described properties of the {01.2} and {01.8} forms.

At this point the circle closes and it becomes increasingly reasonable to think of a competitive cooperation among homo-epitaxy rules and twinning laws in calcite. On the other hand, both the lattice geometry calculation and the 2D-LC energy hypothesis make us think that the {01.0} prism can be excluded from homo-epitaxy. This further confirms that even the {01.0} twinning law is meaningless.

## Figures and Tables

**Table:** 

	Rank	(01.8)_Cal_	(01.2)_Cal_	Linear and area misfit (%)	Notes
Vectors (Å)	1*a*	[010] = 4.9896	[100] = 4.9896	0	Rectangular cell
		1/3 × [ 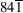 ] = 12.8498	2/3 × [ 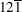 ] = 12.7494	−0.79	
Area (Å^2^) and multiplicity		64.12 (1×)	63.61 (2×)	−0.79	

**Table:** 

	(01.8)_Cal_	(10.4)_Cal_	Linear and area misfit (%)	Notes
Vectors (Å)	[010] = 4.9896	[010] = 4.9896	0	Rectangular cell
	2/3 × [ 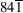 ] = 25.6996	[ 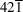 ] = 24.3090	−5.72	
Area (Å^2^) and multiplicity	128.23 (2×)	121.29 (3×)	−5.72	

**Table:** 

	(01.2)_Cal_	(10.4)_Cal_	Linear and area misfit (%)	Notes
Vectors (Å)	[010] = 4.9896	[010] = 4.9896	0	Rectangular cell
	4/3 × [ 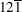 ] = 25.4988	[ 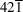 ] = 24.3090	−4.89	
Area (Å^2^) and multiplicity	127.23 (4×)	121.29 (3×)	−4.89	

**Table:** 

	(10.4)_Cal_	(00.1)_Cal_	Linear and area misfit (%)	Notes
Vectors (Å)	1/3 × [ 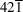 ] = 8.1030	[210] = 8.6422	6.65	Rectangular cell
	[010] = 4.9896	[010] = 4.9896	0	
Area (Å^2^) and multiplicity	40.43 (1×)	43.12 (2×)	6.65	
